# Knockout of Putative Tumor Suppressor Aldh1l1 in Mice Reprograms Metabolism to Accelerate Growth of Tumors in a Diethylnitrosamine (DEN) Model of Liver Carcinogenesis

**DOI:** 10.3390/cancers13133219

**Published:** 2021-06-28

**Authors:** Natalia I. Krupenko, Jaspreet Sharma, Halle M. Fogle, Peter Pediaditakis, Kyle C. Strickland, Xiuxia Du, Kristi L. Helke, Susan Sumner, Sergey A. Krupenko

**Affiliations:** 1 Department of Nutrition, University of North Carolina, Chapel Hill, NC 27599, USA; natalia_krupenko@unc.edu (N.I.K.); susan_sumner@unc.edu (S.S.); 2 Nutrition Research Institute, University of North Carolina, Kannapolis, NC 28081, USA; sharmaj@email.unc.edu (J.S.); halle_fogle@unc.edu (H.M.F.); ppdiaditakis@lygenesis.com (P.P.); 3 Department of Pathology, Duke University, Durham, NC 27708, USA; kyle.strickland@duke.edu; 4 Department of Bioinformatics & Genomics, UNC Charlotte, Charlotte, NC 28223, USA; Xiuxia.Du@uncc.edu; 5 Department of Comparative Medicine, Medical University of South Carolina, Charleston, SC 29425, USA; helke@musc.edu

**Keywords:** ALDH1L1, mouse knockout, folate metabolism, liver cancer, diethylnitrosamine (DEN) carcinogenesis, metabolomics

## Abstract

**Simple Summary:**

Cancers often loose the enzyme of folate metabolism ALDH1L1. We proposed that such loss is advantageous for the malignant tumor growth and tested this hypothesis in mice proficient or deficient (gene knockout) in ALDH1L1 expression. Liver cancer in both groups was induced by injection of chemical carcinogen diethylnitrosamine. While the number of tumors observed in ALDH1L1 proficient and deficient mice was similar, tumors grew faster and to a larger size in the knockout mice. We conclude that the ALDH1L1 loss promotes liver tumor growth without affecting tumor initiation or multiplicity. Accelerated growth of tumors lacking the enzyme was linked to several metabolic pathways, which are beneficial for rapid proliferation.

**Abstract:**

Cytosolic 10-formyltetrahydrofolate dehydrogenase (ALDH1L1) is commonly downregulated in human cancers through promoter methylation. We proposed that ALDH1L1 loss promotes malignant tumor growth. Here, we investigated the effect of the *Aldh1l1* mouse knockout (*Aldh1l1^−/−^*) on hepatocellular carcinoma using a chemical carcinogenesis model. Fifteen-day-old male *Aldh1l1* knockout mice and their wild-type littermate controls (*Aldh1l1^+/+^*) were injected intraperitoneally with 20 μg/g body weight of DEN (diethylnitrosamine). Mice were sacrificed 10, 20, 28, and 36 weeks post-DEN injection, and livers were examined for tumor multiplicity and size. We observed that while tumor multiplicity did not differ between *Aldh1l1^−/−^* and *Aldh1l1^+/+^* animals, larger tumors grew in *Aldh1l1^−/−^* compared to *Aldh1l1^+/+^* mice at 28 and 36 weeks. Profound differences between *Aldh1l1^−/−^* and *Aldh1l1^+/+^* mice in the expression of inflammation-related genes were seen at 10 and 20 weeks. Of note, large tumors from wild-type mice showed a strong decrease of ALDH1L1 protein at 36 weeks. Metabolomic analysis of liver tissues at 20 weeks showed stronger differences in *Aldh1l1^+/+^* versus *Aldh1l1^−/−^* metabotypes than at 10 weeks, which underscores metabolic pathways that respond to DEN in an ALDH1L1-dependent manner. Our study indicates that *Aldh1l1* knockout promoted liver tumor growth without affecting tumor initiation or multiplicity.

## 1. Introduction

Hepatocellular carcinoma (HCC) is the most common primary liver malignancy, with a high mortality rate [1,2]. HCC is commonly associated with chronic inflammation caused by alcohol consumption, non-alcoholic fatty liver disease, exposure to toxic compounds, or viral infection [3]. However, precise molecular mechanisms of liver carcinogenesis, as well as mechanisms promoting the growth of aggressive liver tumors, are not fully understood. To develop targeted therapeutic strategies, a more thorough understanding of the genetic alterations and metabolic derangements of HCC is needed. To this end, numerous studies have evaluated the gene expression profile of HCCs (reviewed in [4,5,6,7]). One study, profiling global gene expression, identified *ALDH1L1* as one of the most under-expressed genes in HCCs compared to normal livers [8]. Furthermore, this study demonstrated that *ALDH1L1* expression was also strongly downregulated in metastatic liver carcinomas compared to normal livers. Another study highlighted the downregulation of *ALDH1L1* as a part of a gene signature for late-stage compared to early-stage HCCs as well as for high-grade compared to low-grade cancers [9]. In line with these findings, it was reported that the decreased expression of ALDH1L1 was associated with poor prognosis in HCC [10].

*ALDH1L1* encodes the cytosolic isoform of 10-formyltetrahydrofolate dehydrogenase, a major enzyme of folate pathways [11] that is highly expressed in the liver, the primary organ of folate metabolism [12]. In the cell, folate coenzymes are involved in numerous biochemical reactions of one-carbon transfer [13,14,15], with central roles in the biogenesis of several amino acids and biosynthesis of nucleotides. Importantly, through the regeneration of methionine from homocysteine, folate metabolism is directly linked to the regulation of cellular methylation since methionine is the precursor of the universal methyl donor, SAM [13,14,15]. Additionally, folate is involved in the regulation of translation in mitochondria [16] as well as in NADPH generation [17]. Higher animals cannot synthesize folate and depend on diet to provide this vitamin. Folate-dependent methylation and nucleotide biosynthesis are especially important for rapidly proliferating cells [18]. Therefore, cancer cells are particularly sensitive to alterations in folate availability or insufficiency of folate metabolism. To support enhanced folate metabolism, malignant tumors commonly overexpress certain folate enzymes [19,20,21]. More specifically to HCC, the folate enzyme MTHFD1L is upregulated in HCC patients, providing a metabolic advantage for tumor growth [22]. Further, regarding the role of folate in HCC, it was observed that the growth rate of HCC-derived cells is reduced after folate withdrawal [22].

ALDH1L1 is not expressed in the vast majority of cancer cell lines because of extensive methylation of the *ALDH1L1* promoter, the mechanism also responsible for the silencing of this gene in malignant human tumors [23,24,25,26,27]. Furthermore, the expression of the enzyme in ALDH1L1-deficient cancer cells evokes an antiproliferative phenotype [12]. This effect is likely due to the interference with de novo purine biosynthesis [28] but may also be caused by the loss of folate-bound one-carbon groups, which are irreversibly removed as CO_2_ in the ALDH1L1-catalyzed reaction [29]. Thus, expression of the enzyme has a broad impact on folate metabolism, including deregulation of methylation [30]. The downregulation of ALDH1L1 expression is found in many cancer types [9], and the loss of the enzyme may confer a selective advantage for rapidly proliferating cells (reviewed in [29]). This hypothesis is further supported by the finding that ALDH1L1 is reversibly downregulated in the S-phase of the cell cycle in NIH3T3 cells, though through a different mechanism (i.e., proteasomal degradation in NIH3T3 cells versus promoter methylation in cancer cells) [31]. In the present study, we investigated whether the loss of ALDH1L1 has an effect on tumor initiation and progression in a DEN model of liver carcinogenesis. We present evidence that, in the absence of this enzyme, liver tumors proliferate faster and grow to a larger size, whereas no apparent effect of ALDH1L1 on tumor initiation was found. Our metabolomic analysis further sheds light on how ALDH1L1 loss provides a metabolic advantage for rapidly proliferating cells.

## 2. Materials and Methods

### 2.1. Animal Experiments

All animal experiments were conducted in strict accordance with the National Institutes of Health’s “Guide for Care and Use of Laboratory Animals” and were approved by the Institutional Animal Care and Use Committee at the Medical University of South Carolina (MUSC), Charleston, South Carolina. Mice were housed in microisolator cages on a 12-h light/dark cycle and allowed access to water and standard rodent chow ad libitum.

### 2.2. Genotyping

Genotyping was carried out by polymerase chain reaction (PCR) of tail lysates obtained using direct PCR (tail) lysis reagent (cat. #101-T) and proteinase K (specific activity >600 U/mL, Thermo Scientific, cat. #EO0491). Primers for genotyping are shown in Table S1. Amplification generated a 199 bp amplicon for the WT allele and a 685 bp amplicon for the mutant allele. Heterozygous males and females were intercrossed to obtain knockout and wild-type littermates.

### 2.3. DEN Administration and Sample Collection

*Aldh1l1^+/+^* and *Aldh1l1^−/−^* male littermates at 14–15 days of age were subjected to a single intra-peritoneal DEN injection at a dose of 20 μg/g body weight [32,33]. DEN was purchased from Sigma (cat. # N0756). After injection, mice were maintained under standard conditions on standard rodent chow for defined periods of 10, 20, 28, and 36 weeks (eight animals per group). After mice were euthanized, body weights were recorded and whole livers were collected for analysis.

### 2.4. Liver Tumor Analysis

The whole liver was carefully removed from euthanized animals and washed with cold PBS. Livers were photographed, their weights were recorded, visible tumor lesions were counted, and the size of large tumors (diameter >5 mm) was measured. Segments of the liver left lateral lobes were placed in OCT medium and fast-frozen for future analysis. Additional liver sections were collected and fixed in 10% formalin. Fixed tissues were embedded in paraffin, and tissue blocks were cut into 5-μm sections. Tissue section slides were stained with H&E and reticulin (reticulin staining kit, Abcam; ab-150684) using standard protocols and evaluated by a board-certified anatomic pathologist using an Olympus BX46 microscope. The number of basophilic foci and nodules of hepatocellular carcinoma were examined and tallied for a single tissue section from each animal.

### 2.5. Immunohistochemical (IHC) Staining and Review

Slides were deparaffinized, rehydrated, and treated with 3% hydrogen peroxide. Antigen retrieval was performed by boiling the sections for 10 min in 0.1 M citrate buffer antigen retrieval solution (pH 6.0). Non-specific antibody binding was blocked using 2% non-fat milk in TBST for 30 min. Sections were probed with primary ALDH1L1-specific in-house polyclonal antibody (1:1000, 4 °C overnight) followed by incubation with anti-rabbit secondary antibody (GE NA934V; 1:250, 1 h at room temperature). The slides were incubated with ABC reagent (Vectastain ABC kit, PK-4000) for another 30 min at room temperature, washed with TBST, and stained with 3,3′-diaminobenzidine (DAB, Vector laboratories SK-4105). Separate liver sections were stained with Ki-67 antibody (Cell Signaling, 9027 S). Cells with positive staining were scored in at least 100 hepatocytes and reported as mean ± SD, and *n* = 5 or more mice were used in each group. Immunostained slides were examined by a board-certified anatomic pathologist. Digital photomicrographs were obtained using an Olympus BX46 microscope at high power (40x), and the number of positive and negative nuclei were counted to determine the percent Ki-67 proliferation index. Photomicrographs of tissue sections stained with CD34 antibody (ab81289) were obtained at 40x magnification in 3 fields of normal liver and 1 to 3 fields in neoplastic foci if present on the slide. Slides were blindly analyzed using ImageJ software (Fiji v.2.0.0; National Institutes of Health, Bethesda, MD, USA. Each image was subjected to color-deconvolution using the built-in vector H-DAB, in which the brown staining of hematoxylin and diaminobenzidine (DAB) is isolated from other colors in the image, and this DAB image was used to calculate the number of stained pixels and % area stained in each image. For statistical purposes, the Mann-Whitney test was used to compare values, and significance was evaluated at the 0.05 level.

### 2.6. Western Blot Assays

Total protein was prepared from flash-frozen liver tissue. Approximately 300 mg liver tissue was minced and homogenized in 1 mL of RIPA buffer with protease and phosphate inhibitors (1:100) (Thermo Scientific, Waltham, MA, USA). Proteins were resolved on SDS polyacrylamide gel electrophoresis in 4–15% gels and then transferred to PVDF membranes (Millipore, Bedford, MA, USA) in transfer buffer containing 10% methanol. Membranes were probed with primary ALDH1L1-specific in-house polyclonal antibody (1:10,000) in Tris-buffered saline with Tween- 20 containing 5% nonfat milk. Horseradish peroxidase-conjugated secondary antibodies were used at 1:5000 dilution, and the signal was assessed with Super Signal West Pico chemiluminescence substrate (Pierce, Rockford, IL, USA).

### 2.7. Real-Time PCR

Total RNA was extracted from frozen liver tissues. cDNA was generated using a reverse transcription kit (Thermo Fisher Scientific-4368814). Real-time PCR was performed using a Realplex4 Mastercycler (Eppendorf, Hauppauge, NY, USA) and SYBR Green PCR master mix (Applied Biosystem, Waltham, MA, USA). The PCR reaction was set up as follows: 20 μL PCR mixture containing 10 μL SYBR Premix EX Taq, 2 μL cDNA (100 ng), 0.4 μL (10 μM) each forward and reverse primer, and 6.8 μL ddH_2_O. The PCR protocol included initial 95 °C melting for 5 min and then 40 cycles (denaturation at 95 °C for 30 s, annealing at 60 °C for 30 s, and elongation at 72 °C for 20 s). Levels of target mRNAs were normalized by the levels of GAPDH as a housekeeping gene. The fold change in mRNA expression was calculated using 2^−ΔΔCt^. The primers for the assay are shown in Table S2.

### 2.8. RT-PCR Data Pretreatment/Transformation

Each gene/protein was standardized by subtracting the mean from the original data and then dividing by the standard deviation so that the mean of the transformed data was 0 and the standard deviation of the transformed data was 1.

### 2.9. Metabolome Analysis

Metabolomics was performed using commercial services from Metabolon (Durham, NC, USA). Individual samples (100–200 mg of flash-frozen tissue) were subjected to methanol extraction then split into aliquots for analysis by ultrahigh performance liquid chromatography/mass spectrometry (UHPLC/MS). A detailed description of the metabolome-related methodology is provided in Supplementary Methods. Additional analysis was performed using SIMCA-p (Umetrics).

### 2.10. Statistical Analysis

Statistical analysis was carried out using Graph Pad Prism VII software. Statistical significance was calculated with a Student’s *t*-test. Multivariate analysis was performed using SIMCA 15.0 (Umetrics, Sartorius Stedim Data Analytics, AB, Umeå, Sweden). Unsupervised principal component analysis (PCA) was used to visualize the differentiation between the study groups, and orthogonal partial least squares discriminate analysis (OPLS-DA) was used to determine the variable influence on projection (VIP) for determining metabolites that contributed to the differentiating profiles.

## 3. Results

### 3.1. Aldh1l1 Knockout Alters Tumor Growth Dynamics in a DEN Model Of Carcinogenesis

**Figure 1:**
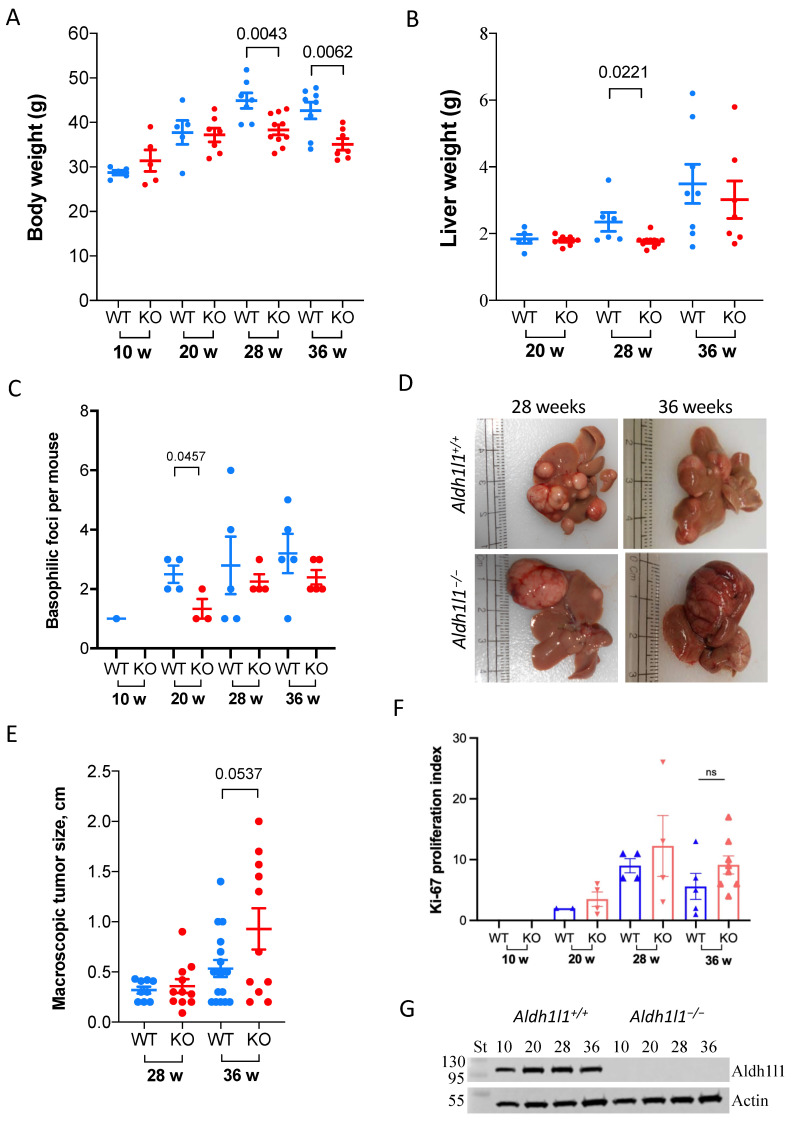
Comparison of WT and *Aldh1l1* KO mice after DEN injection. Mouse body (**A**) and liver (**B**) weight. (**C**) Number of basophilic foci from microscopic evaluation of liver tissue cross-sections. (**D**) Representative liver images of DEN-treated mice (macroscopic tumors are clearly visible as large lesions on the surface). (**E**) Sizes of macroscopic tumors in each animal group. (**F**) Ki-67 proliferation index calculated from immunostaining (as described in Materials and Methods, Section 2.5). ns: Not significant (**G**) Levels of ALDH1L1 protein in the liver at different time points post-DEN treatment (Western blot assay with ALDH1L1-specific antibody; 20 μg of total protein was used for each sample; actin is shown as loading control).

The formation of tumors followed single intraperitoneal DEN injections of 15-day-old *Aldh1l1^−/−^* (knockout, KO) and *Aldh1l1^+/+^* (wild-type, WT) male mice [32,33]. In our experimental model, 100% of both KO and WT mice developed large tumors at 36 weeks; therefore, experiments were not extended beyond this time point. Similar timing of HCC development in C57/Bl6 mice with a single DEN injection at 2 weeks of age was observed in other studies [34,35]. We found that at 28 and 36 weeks after DEN injection, *Aldh1l1^−/−^* mice had reduced body weight compared to wild-type mice, with a 20% reduction at 28 weeks (*p* = 0.0043) and a 25% reduction at 36 weeks (*p* = 0.0062), respectively (Figure 1A). We previously reported similar differences in body weights for *Aldh1l1^−/−^* versus *Aldh1l1^+/+^* untreated mice [36]. Of note, DEN-treated mice had noticeably higher body weights than untreated mice, which is in agreement with previously reported studies [35]. Liver weights were also different between *Aldh1l1^−/−^* mice and *Aldh1l1^+/+^* mice at these time points, though this difference was statistically significant only at the 28-week time point (Figure 1B). If normalized to body weight, the difference in liver size between DEN-treated KO and WT mice was reduced but still observed at 28 weeks (Figure S1).

Histologic analysis of DEN-exposed liver demonstrated hepatic neoplasia, first visible as round microscopic foci of basophilic hepatocytes with indistinct cell borders, nuclear hyperchromasia, nuclear atypia, and an increased nuclear-to-cytoplasm ratio. These basophilic foci were present as early as 20 weeks in the KO and 10 weeks in WT mice (Figure 1C and Figure S2). Vascular involvement, which could be highlighted using reticulin staining, was present in both the KO and WT mice as early as 20 weeks (Figure S2). Larger tumor nodules (macroscopic tumors with at least one linear dimension >0.5 cm) were observed starting at 20 weeks in *Aldh1l1* KO and 28 weeks in WT mice (Figure S3). At both 28 and 36 weeks post-injection, macroscopic tumors in *Aldh1l1^−/−^* mice were larger than those observed in *Aldh1l1^+/+^* mice (Figure 1D,E). At 36 weeks the average gross tumor diameter was 0.93 cm for KO mice and 0.53 cm for the WT mice (*p* = 0.0537). Ki-67 proliferative index of neoplastic foci was similar for both animal groups. Although at the 36-week time point *Aldh1l1* KO mice demonstrated a decreased number of microscopic lesions (nodules plus basophilic foci) compared to WT mice (*p* = 0.0340), our analysis of tissue cross-sections suggested that invasive tumors (as well as precursors) developed in the KO and WT mice at approximately the same frequency. The analysis of the Ki-67 proliferation index also suggested enhanced cellular proliferation in the livers of DEN-treated *Aldh1l1^−/−^* mice compared with *Aldh1l1^+/+^* mice, though the data did not reach statistical significance (Figure 1F).

### 3.2. Large Tumors in DEN-Treated Wild-Type Mice Lost ALDH1L1 Protein

**Figure 2:**
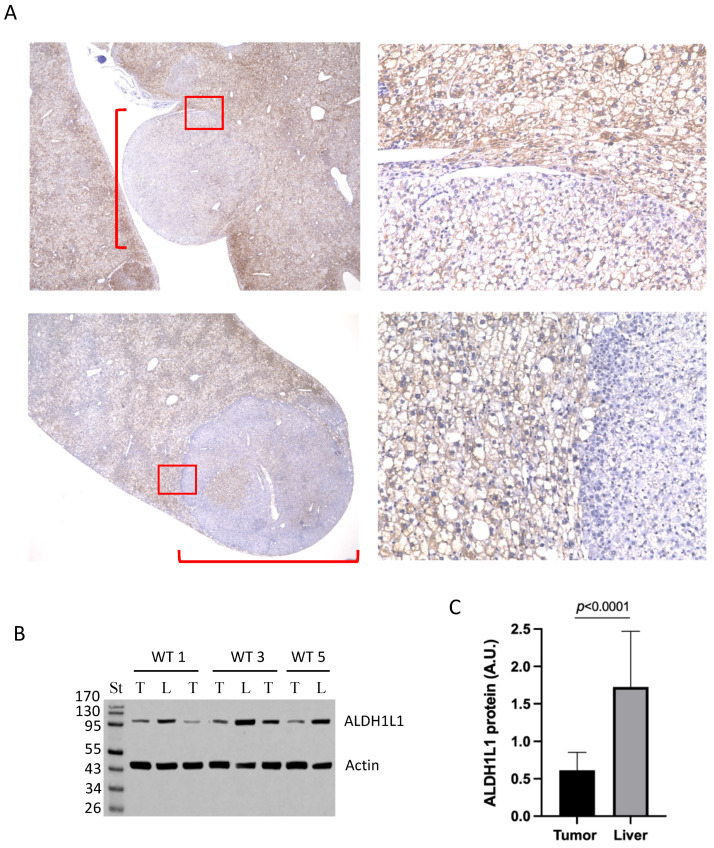
Levels of ALDH1L1 protein in large tumors grown in *Aldh1l1^+/+^* mice. (**A**) Immunohistochemical staining of large tumors (36 weeks post-DEN injection) in *Aldh1l1^+/+^* mice with ALDH1L1-specific antibody. Large tumors (from two mice) are marked with a *red* square bracket (2x magnification). Areas marked with *red* squares are shown at higher magnification (20×) on the right panels. (**B**) Western blot assay of large tumors (T) and normal liver tissues (L) of *Aldh1l1^+/+^* mice (3 mice, WT1, 3, and 5) with ALDH1L1-specific antibody. (**C**) Calculated intensities of ALDH1L1 bands normalized to actin (Image J), arbitrary units (A.U.). Experiments for all samples were repeated three times (technical replicates); the calculation includes all replicates (biological and technical; *p* < 0.0001).

*ALDH1L1* was reported as one of the most under-expressed genes in HCC [8]. To test whether the *Aldh1l1* gene expression changed in response to DEN injection, we evaluated ALDH1L1 protein levels by Western blot assays at 10–36 weeks post-treatment. No overall changes in the levels of ALDH1L1 were observed in the livers of DEN-exposed *Aldh1l1^+/+^* mice in these experiments (Figure 1G and Figure S7). We further evaluated protein levels of ALDH1L1 in large tumors. Tumors were identified grossly as rounded nodular growths upon serial sectioning of liver tissue (Figure 2A). Histologic evaluation showed that neoplastic foci were composed of cells that were cytologically distinct from normal background hepatocytes, exhibiting enlarged, hyperchromatic nuclei with coarse chromatin. Tumor nodules also demonstrated architectural abnormalities, such as an expansion of the sinusoids, a pushing border, and loss of reticulin by special staining. Intravascular extension was also identified in a number of cases. Histochemical staining with ALDH1L1-specific antibody showed that in wild-type mice, large tumors lost the ALDH1L1 protein, compared to the rest of the liver (Figure 2A).

We further evaluated by Western blot assays the levels of ALDH1L1 protein in macroscopic tumors collected from wild-type mice. In these experiments, macroscopic tumors were visually identified as large rounded nodular growths protruding from liver lobes and were distinctly different from typical liver tissues. We analyzed five such tumors from three *Aldh1l1^+/+^* mice as well as seemingly unaffected liver tissues from the same animals. Western blot assays showed that levels of ALDH1L1 protein were about three-fold lower in tumors compared to liver tissues (Figure 2B,C). This difference was highly statistically significant (*p* < 0.0001, Figure 2C).

### 3.3. Vascular Density in Livers of Aldh1l1^−/−^ and Aldh1l1^+/+^ Mice

Vascular density was evaluated using immunohistochemical staining for CD34 [37] (Figure S4). At all time points, median vascular density was measured as follows: normal liver in WT mice 0.56% (95% CI 0.35–0.81), normal liver in KO mice 0.54% (95% CI 0.29–0.79), neoplastic liver in WT mice 7.8% (95% CI 6.5–9.6), and neoplastic liver in KO mice 5.8% (95% CI 4.4–9.0). Vascular density was increased in neoplastic nodules compared to normal liver in both WT (*p* < 0.0001) and KO mice (*p* < 0.0001), but the vascular density was not significantly different between WT and KO mice for normal liver (*p* = 0.50) or neoplastic nodules (*p* = 0.17).

### 3.4. Expression of Cancer-Related Genes in DEN-Treated Mice

**Figure 3:**
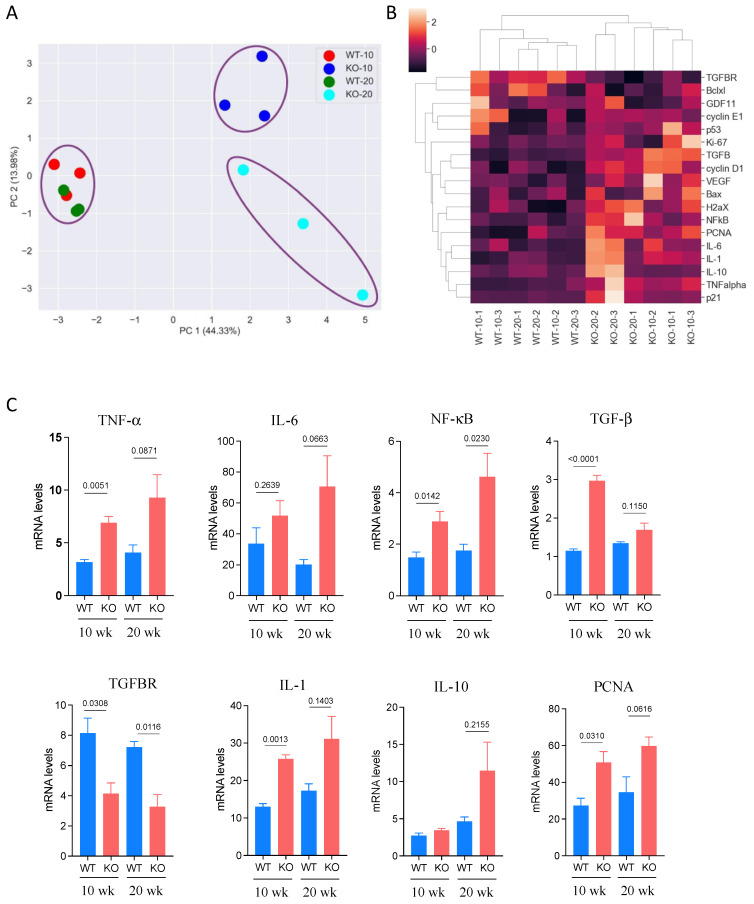
Expression of inflammation-related genes in DEN-treated livers analyzed by RT-PCR. (**A**) PCA for *Aldh1l1^+/+^* (WT) and *Aldh1l^−/−^* (KO) mice carried out using the Python sklearn.decomposition package. The first and second PC of the standardized data explained 44% and 14% of the total variance, respectively. Time points post-DEN injection (10 and 20 weeks) are indicated. (**B**) Standardized RT-PCR data from data pretreatment displayed as a heatmap. The heatmap was produced using the Python seaborn package. Three mice per group (four groups, two genotypes/two time points) were analyzed. (**C**) Relative levels (RT-PCR) of specific targets.

We previously reported that *Aldh1l1* knockout affected the expression of numerous genes relevant to inflammation [36]. Based on the reported role of pro-inflammatory genes in HCC, we selected a subset of genes from a previously evaluated panel to assess their expression in the livers of *Aldh1l1^−/−^* and *Aldh1l1^+/+^* mice 10 and 20 weeks post-DEN injection. The expression of 18 genes relevant to the development of HCC was assessed by RT-PCR (Figure 3). PCA showed that based on the expression of these genes, there was good separation between the WT and KO groups (Figure 3A). There was also a clear separation between the two time points for the KO groups, with marginal differences between the groups for WT mice (Figure 3A). The heat map (Figure 3B) illustrates the strong difference between WT and KO mouse livers at both time points with regard to the expression of the evaluated genes. In these experiments, profound differences between WT and *Aldh1l1* KO mice were seen for NF-κB, IL-6, IL-10, TNF-α, PCNA, TGF-β, and TGFBR (Figure 3C). The elevation of PCNA observed in these experiments (Figure 3C) is supportive of the ALDH1L1 role in proliferation regulation.

### 3.5. Aldh1l1^−/−^ Mice Demonstrate an Altered Metabolic Response to DEN Compared To Wild-Type Mice

#### 3.5.1. Overall Metabolic Changes

**Figure 4:**
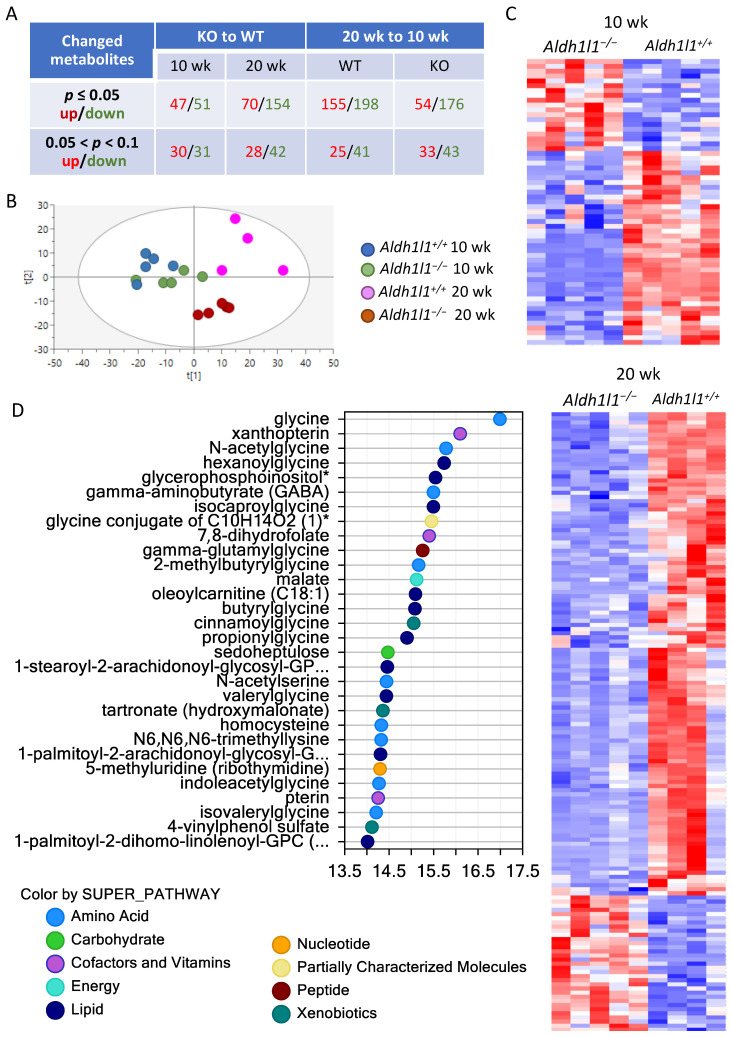
Analysis of the liver metabolomes of *Aldh1l1^+/+^* and *Aldh1l1^−/−^* male mice 10 and 20 weeks after DEN injection. (**A**) Summary of metabolome analysis. (**B**) PCA visualization of metabolomic profiles. (**C**) Heatmap representation of the metabolite comparison between *Aldh1l1^+/+^* and *Aldh1l1^−/−^* male mice 10 and 20 weeks after DEN injection (performed with Qlucore Omics Explorer v.3.4 software, Qlucore, Lund, Sweden; data were filtered by *p*-value ≤ 0.05). (**D**) Biochemical importance plot (Random Forest Analysis).

To understand the metabolic basis for the promotion of tumor proliferation in Aldh1l1 KO mice, we performed a metabolomic analysis of liver samples at 10 and 20 weeks post-DEN injection. In our study, hepatic lesions at these time points were limited to basophilic foci, with no macroscopic tumors observed in the entire WT group or the vast majority of the KO group (Supplementary Figures S2 and S3). Thus, we were able to analyze metabolic changes before the malignant tumors were formed. This analysis showed strong differences between metabotypes of KO and WT mice at both time points as well as profound changes for both genotypes between the 10- and 20-week time points (Figure 4A and Table S3). The principal component analysis (PCA) visualization showed that the differences in metabotypes between KO and WT mice were more prominent at 20 weeks than the differences between KO and WT at 10 weeks (Figure 4B, clustering of 20-week-groups is evident). This trend was further exemplified by heatmaps generated using Qlucore software (Figure 4C). The variable importance to projection (VIP) scores for KO vs. WT at 10 weeks and KO vs. WT at 20 weeks further showed that the repertoire of metabolites separating KO from WT was different at the 10- vs. 20-week time points (Table S4). Several major cellular pathways contributed to the separation between *Aldh1l1^+/+^* and *Aldh1l1^−/−^* mice in their metabolic responses to DEN (Figure 4D), including the pathways of folate, glycine, lipid, and carbohydrate metabolism as well as metabolites involved in the regulation of redox homeostasis.

#### 3.5.2. Folates and Related Metabolites

**Figure 5:**
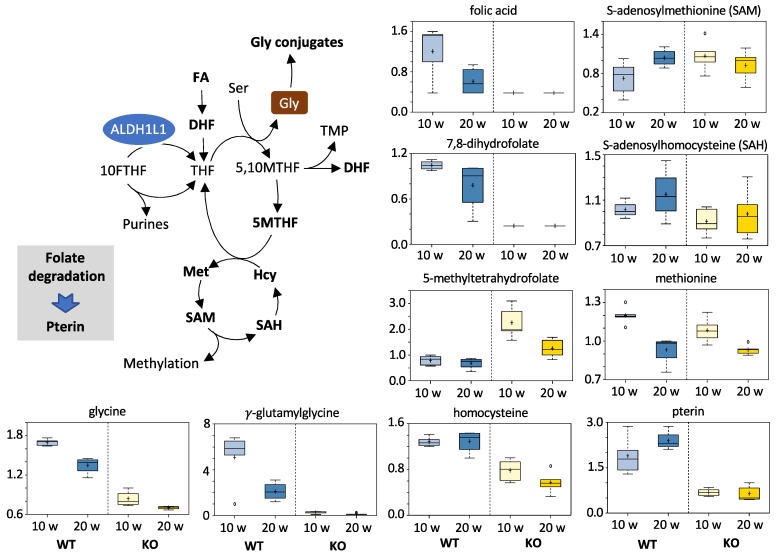
Comparison of the folate pathway metabolites between animal groups. Schematic depicts folate metabolism as it is relevant to listed compounds. Folate degradation is proposed as the source of pterin. *Y*-axes of individual metabolite box plots represent scaled intensity. Measurements for each biochemical in the original scale were rescaled to set the median equal to 1. Abbreviations: FA, folic acid; DHF, 7,8-dihydrofolate; THF, tetrahydrofolate; 5MTHF, 5-methyltetrahydrofolate; 5,10MTHF, 5,10-methylene-THF; 10FTHF, 10-formyl-THF.

Folate (folic acid) and 7,8-dihydrofolate (DHF) were strongly depleted in the livers of KO mice compared to WT mice at both time points (2.86-fold, *p* = 0.0006 and 4.35-fold, *p* = 0.0000 at 10 weeks and 1.49-fold, *p* = 0.1405 and 2.94-fold, *p* = 0.0000 at 20 weeks, folate and DHF, respectively). A similar effect was observed previously in the KO versus WT mice [36]. Additionally, higher 5-methyltetrahydrofolate (5MTHF) was measured in KO animals compared to WT groups at both 10 and 20 weeks (2.84-fold, *p* = 0.0010 and 1.87-fold, *p* = 0.0081 for the 10- and 20-week time points, respectively). Of note, at 20 weeks, differences in folate pools between KO and WT mice became significantly smaller. In addition, in agreement with our previous report, levels of the histidine degradation intermediate FIGLU (formiminoglutamate) were elevated in KO mice, though these values did not reach statistical significance (Table S3). Of note, histidine was noticeably decreased in WT mice at 20 weeks compared to 10 weeks post-DEN treatment (Table S3). Interestingly, levels of homocysteine were lower in the knockout mice at both time points, with stronger differences observed at 20 weeks post-DEN injection. Levels of two amino acids, whose biosynthesis is linked to homocysteine, were also altered in our experiments: methionine was decreased in both WT and KO mice at 20 weeks compared with 10 weeks, while cysteine was decreased in the KO group only at 20 weeks compared with 10 weeks (Table S3). (See Figure 5 and Table S3).

#### 3.5.3. Acylglycine Conjugates

In agreement with our previous studies, the present analysis showed significantly lower levels of glycine (2-fold) and numerous acylglycine conjugates in KO compared to WT mice at both 10 weeks (1.82- to 7.69-fold) and 20 weeks (1.14- to 8.33-fold) post-DEN injection. The only exception was 3,4-methylene heptanoylglycine, which was higher in KO mice. This, however, could be explained by the origin of this metabolite from microbiota [38]. Levels of glycine were about two-fold lower in KO versus WT mice at both time points. This phenomenon is consistent with the role of ALDH1L1 in generating THF, which is required for glycine production from serine [36]. Levels of glycine were also decreased by about 20% at 20 weeks compared to 10 weeks for both genotypes (Figure 5 and Table S3). Interestingly, the same phenomenon was observed for several acylglycines in the livers of wild-type mice, with their levels being decreased more than two-fold between 10 and 20 weeks. (See Figure S5).

#### 3.5.4. Monoacylglycerols and Fatty Acids

**Figure 6:**
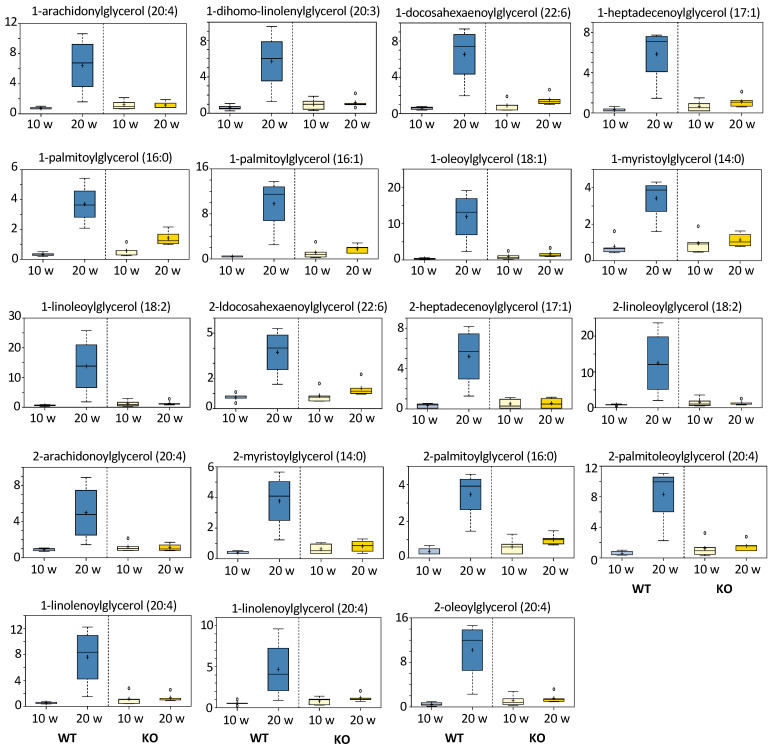
Effect of DEN on monoacylglycerols in the livers of *Aldh1l1^+/+^* (WT) and *Aldh1l1^−/−^* (KO) mice. *Y*-axes of individual metabolite box plots represent scaled intensity. Measurements for each biochemical in the original scale were rescaled to set the median equal to 1.

**Figure 7:**
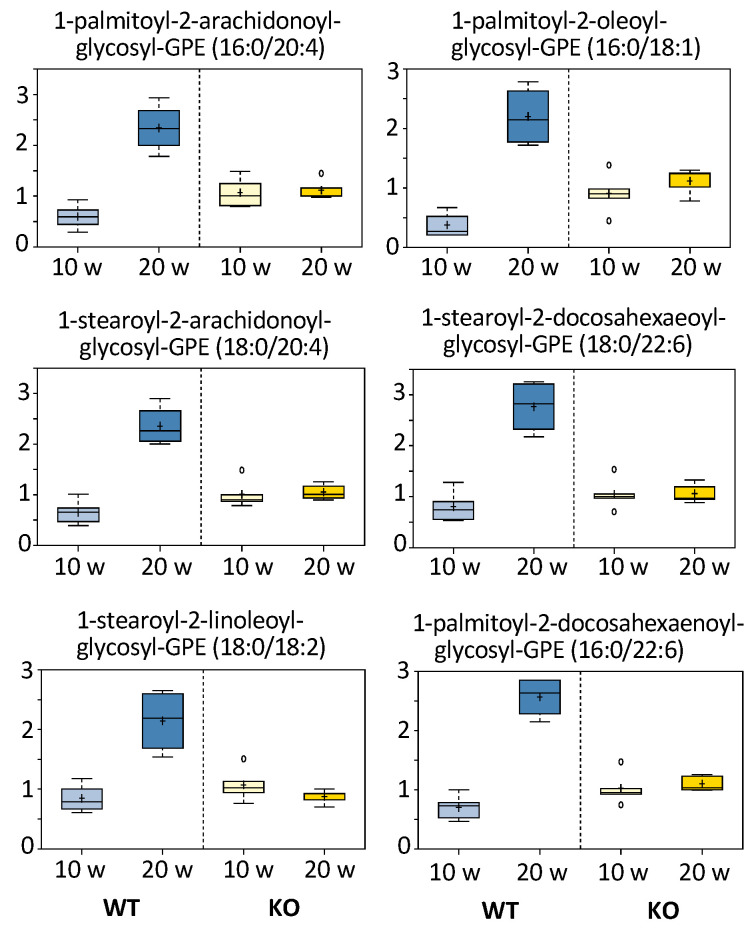
Comparative levels of glycosyl-GPE metabolites in the livers of DEN treated *Aldh1l1^+/+^* (WT) and *Aldh1l1^−/−^* (KO) mice. *Y*-axes of individual metabolite box plots represent scaled intensity. Measurements for each biochemical in the original scale were rescaled to set the median equal to 1.

The most noticeable difference in metabolites between 10 and 20 weeks post-DEN injection was a strong elevation of all measured monoacylglycerols (total of 19) in WT mice at 20 weeks (Figure 6). Notably, such changes did not take place in KO mice. Similarly, levels of free fatty acids were markedly elevated in WT but not KO mice at 20 weeks (Figure S6). The simultaneous elevation of monoacylglycerides and free fatty acids indicated more active hydrolysis of diacylglycerides (most likely in already initiated liver cells) in WT mice than in KO mice. Interestingly, six measured glycosyl-GPE (glycerophosphoethanolamine) species showed the same pattern as monoacylglycerols in WT versus KO mice (Figure 7). Non-glycosylated GPE metabolites were similar between WT and KO mice and showed a decrease in both groups between 10 and 20 weeks after DEN injection (Table S3). (See Figure 6 and Figure S6).

#### 3.5.5. Carbohydrates 

**Figure 8:**
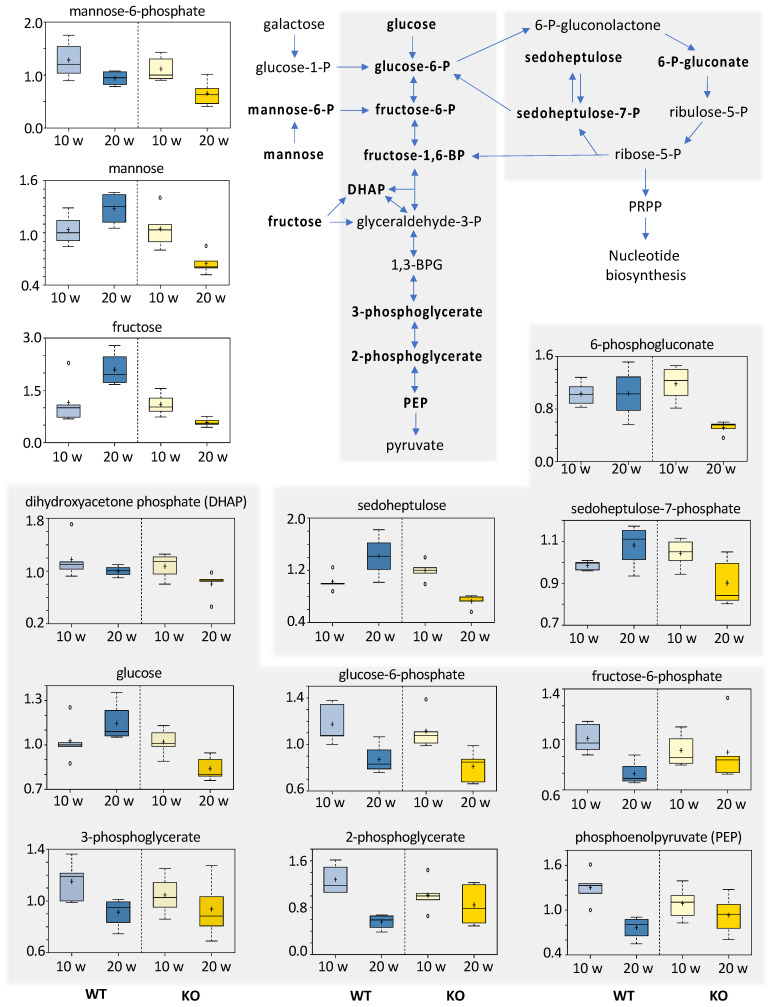
Effect of DEN on carbohydrates in WT and *Aldh1l1* KO mice. Diagram schematically depicts glycolysis (highlighted in *pink*) and the pentose phosphate pathway (highlighted in *light green*). Metabolites identified by metabolomics are shown in *boldface* in the diagram. *Y*-axes of individual metabolite box plots represent scaled intensity. Measurements for each biochemical in the original scale were rescaled to set the median equal to 1.

Significantly lower levels of several sugars were seen in KO mice when they progressed from 10 to 20 weeks post-DEN injection. Specifically, the level of glucose at 20 weeks was 1.37-fold lower in KO than in WT mice (*p* = 0.0007). In addition, there was a significant decrease (1.22-fold, *p* = 0.0122) in glucose content in KO but not WT mice at 20 weeks compared to 10 weeks post-DEN injection. This could be associated with the increased rate of glycolysis, a known phenomenon in cancer cells (reviewed in [39,40]). In support of this interpretation, several intermediates of glycolysis were also decreased between the 10- and 20-week time points (Figure 8). However, the degree of reduction of these intermediates was similar in both WT and KO mice. In contrast to this observation, another glucose-related metabolic branch, the pentose phosphate pathway (PPP), was altered in WT and KO mice by DEN injection: three intermediates of the PPP (6-phosphogluconate, sedoheptulose-7-phosphate, and sedoheptulose) were decreased between 10 and 20 weeks in KO but not in WT mice. We interpreted this effect as the activation of the PPP in KO mice, and the phenomenon might explain the early growth of larger tumors in this group. Specifically, phosphoribosyl pyrophosphate (PRPP), the precursor of de novo nucleotide biosynthesis, is generated from the PPP metabolite ribose-5-phosphate. Of note, the de novo nucleotide biosynthesis is particularly active in rapidly proliferating cells [41]. In support of our conclusion, similar dependence between metabolites of PPP and HCC was observed in another mouse model [42]. (See Figure 8).

#### 3.5.6. Redox Homeostasis Metabolites 

**Figure 9:**
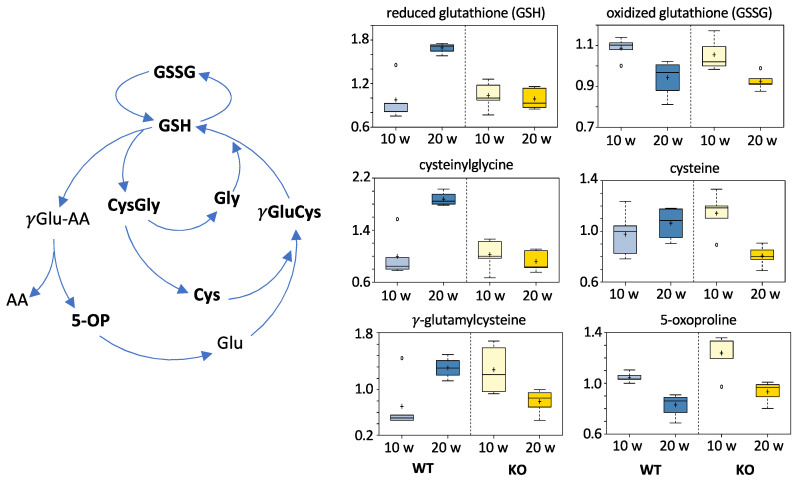
Glutathione cycle and comparative levels of relevant metabolites assigned by metabolomic analysis. Metabolites important to differentiation of the WT and KO mice are shown in *boldface* in the diagram. *Y*-axes of individual metabolite box plots represent scaled intensity. Measurements for each biochemical in the original scale were rescaled to set the median equal to 1.

The metabolomic analysis also indicated that the liver cells of KO mice might experience stronger oxidative stress than the cells of WT mice. This conclusion is based on significantly lower levels of GSH, cysteine, and cysteinylglycine at 20 weeks post-DEN injection. Of note, oxidized glutathione (glutathione disulfide) was not different between WT and KO mice. (See Figure 9).

## 4. Discussion

Numerous reports demonstrating that the major folate enzyme ALDH1L1 is strongly and ubiquitously downregulated in human cancers have led to a hypothesis that the protein could be a putative tumor suppressor (reviewed in [29]). Findings that the downregulation of *ALDH1L1* is linked to the methylation of the CpG island in the gene promoter provided further support for such function [23,24,25,26,27]. Regarding the role of the protein as a tumor suppressor, our cell culture studies demonstrated that forced expression of ALDH1L1 in ALDH1L1-deficient cancer cells produces strong antiproliferative effects, including cell cycle arrest and apoptosis [12,43]. Of note, re-expression of ALDH1L1 in cancer cells activates several downstream antiproliferative signaling pathways, most notably the tumor suppressor p53 and the pro-apoptotic Bid [28,44,45,46,47,48,49]. However, if ALDH1L1 functions as a tumor suppressor, it could be expected that its loss enhances tumorigenesis. We investigated this putative function of the protein by comparing malignant tumor growth in wild-type and *Aldh1l1* knockout mice using the DEN model of liver carcinogenesis. We recently reported that *Aldh1l1* knockout in mice does not lead to any evident phenotype but produces strong metabolic alterations in the liver, evoking metabolic symptoms of functional folate deficiency [36].

Our data indicate that the loss of ALDH1L1 does not have an effect on liver tumor multiplicity in the DEN model but instead leads to more rapid growth of larger tumors. These findings suggest that ALDH1L1 does not suppress carcinogenesis per se but displays an antiproliferative effect on transformed cells at the stage when they undergo rapid expansion to form larger tumors. The role of ALDH1L1 as the regulator of tumor proliferation is in agreement with previous findings that the expression of this enzyme oscillates during the cell cycle, being the lowest in the S-phase of actively proliferating cells [31]. In further agreement with its role as a proliferation regulator rather than a tumorigenesis suppressor, *Aldh1l1* KO mice did not develop spontaneous tumors even at an older age (up to 2 years of age). This is in contrast with another folate-related enzyme, glycine N-methyltransferase (GNMT), which displays several physiological characteristics that mirror those of ALDH1L1. Although GNMT is not involved in folate metabolism directly, its catalysis is regulated by the binding of 5-methyl-THF [50]. Similar to ALDH1L1, GNMT is highly expressed in the liver (comprising up to 3% of the total cytosolic protein in hepatocytes [51]) but ubiquitously downregulated in many cancers, including HCC [52]. GNMT is expressed at a low level in the majority of cancer cell lines and produces an antiproliferative effect, though to a lesser extent than does ALDH1L1 [52]. However, knockout of GNMT in mice leads to spontaneous hepatocellular carcinoma at the age of 8–12 months [53]. Overall, our data imply that, while the role of ALDH1L1 in tumorigenesis has yet to be established, the protein may regulate proliferation in already initiated cells. Importantly, in line with this conclusion, in our study, large DEN tumors developed in wild-type mice with downregulated ALDH1L1 expression, the phenomenon underscoring the selective advantage of the protein loss for developing advanced tumors.

HCC is an inflammation-linked cancer featuring infiltration of the liver with inflammatory cells, which secrete cytokines and chemokines [54,55]. The NF-κB signaling pathway plays a crucial role in liver inflammatory responses by controlling the expression of an array of growth factors and cytokines [56]. One of these cytokines, IL-6, is considered a key regulator of HCC, with its high levels associated with this cancer progression [55,56]. IL-6 is also highly elevated in response to DEN injection, while the loss of this cytokine inhibits DEN-induced liver tumors [57,58]. In our study, in agreement with the tumor-promoting effect of the ALDH1L1 loss, both NF-κB and IL-6 were strongly elevated in *Aldh1l1* KO livers compared to WT livers. Other inflammation-related genes elevated in *Aldh1l1* KO (IL-1, IL-10, TNFα, and TGFβ) were also linked to HCC [54,55,56]. The elevation of this set of inflammation markers in the *Aldh1l1* KO could be a common tumor-promoting response in the DEN model since a similar effect was seen with a high-fat diet, which also promotes DEN-induced tumors [59]. Overall, our study indicates that the loss of ALDH1L1 not only contributes to accelerated cellular proliferation but also promotes an inflammatory response in the liver favorable to HCC progression.

Metabolomics, the global analysis of small molecule metabolites, can provide critical information about the cancer state and is increasingly used in cancer research [60]. Metabolomic analysis offers further support for the idea that ALDH1L1 contributes more control at a later stage of tumor progression. Thus, ALDH1L1-proficient and deficient livers have more similar metabolic profiles at 10 weeks than at 20 weeks post-DEN injection. Our data indicate that the most profound differences in metabolite changes over time between WT and *Aldh1l1* KO mice were in lipid metabolism. This is not surprising since numerous reports have highlighted lipid metabolome changes in HCC as one of the key events [61,62,63,64,65]. Other major pathways affected by DEN carcinogenesis that also respond to the loss of *Aldh1l1* include glycolysis, PPP, and bile acid biosynthesis. Changes in these pathways were previously linked to hepatocellular carcinogenesis [42,65,66,67,68,69]. Numerous metabolomic studies also attempted to identify specific metabolites that would define the metabolic signature of liver carcinogenesis and HCC [64,69,70,71,72,73]. One of these studies, performed in tumor and normal liver samples from 31 patients, highlighted seven metabolites (glucose, glycerol 3- and 2-phosphate, malate, alanine, myo-inositol, and linoleic acid) that decreased in HCC compared to normal samples [74]. Other studies based on metabolomic analysis of serum or urine as well as liver tissues of HCC patients pointed to additional metabolites, which could serve as markers of hepatocarcinogenesis. Of note, changes in the levels of several of those metabolites (N^1^-acetylspermidine, glycocholic acid, 7-methylguanine [65], serine, glycine, linoleic acid [75], hippurate, taurocholate, glycocholate, phytosphingosine, and palmitic acid [69]) in response to DEN exposure in *Aldh1l1* KO mice compared to WT mice are in agreement with the effect of enzyme loss on tumor proliferation.

In contrast to studies of HCC in humans, metabolomic studies of the DEN model of carcinogenesis are scarce. A recent report has shown that DEN-treated rats had decreased levels of GSH in the liver and of glucose, glycerol, and PUFA in the serum [76]. Treatment with an anti-inflammatory drug or the chemotherapeutic 5-fluorouracil in this study resulted in a strong elevation of these metabolites, indicating that the decrease is a likely marker of DEN-induced carcinogenesis. Accordingly, in our study, a stronger decrease of these metabolites in DEN-treated *Aldh1l1* KO versus DEN-treated WT mice is in agreement with the tumor-promoting effect of the *Aldh1l1* loss. In fact, aberrant glucose, lipid, and GSH metabolism is likely a general characteristic of DEN-induced carcinogenesis [77]. Additional metabolites representing a potential signature of DEN carcinogenesis [78], were altered by the ALDH1L1 status. Contrary to our findings, increased GSH levels in DEN-treated mice were previously observed in a study examining the role of lipogenesis in liver tumorigenesis [79]. It was concluded that improved antioxidant defense in mice lacking lipogenesis contributes to the phenotype of increased liver tumorigenesis. Metabolomic analysis in that study, however, was performed at a much later time point (30 weeks post-DEN injection), which might contribute to the observed differences in GSH levels between our studies. Furthermore, we observed an effect on tumor size but not tumor multiplicity, which is also a clear difference between the two studies. Overall, specific metabolic effects in tumorigenesis and the extent and direction of metabolic reprogramming may be highly dependent on the genetic drivers involved. Therefore, it is not surprising that deregulation of folate pathways versus the loss of lipogenesis mediated the tumorigenic effect of DEN in different ways. In another study, it was observed that GSH was elevated over time in both DEN-treated and untreated rats [77], and we observed this phenomenon in our experiments in WT but not *Aldh1l1* KO mice. Thus, the possibility cannot be excluded that the decreased GSH in *Aldh1l1* KO mice is an indication of enhanced ROS removal, which could be beneficial for cancer cell survival.

Folate metabolism influences a large number of cellular pathways, but it is not clear how these pathways, especially those relevant to ALDH1L1, are linked to DEN-initiated responses. ALDH1L1 catalysis is expected to directly affect de novo purine biosynthesis and the mitochondrial translation, pathways requiring 10-formyl-THF, as well as reactions requiring THF. The downstream effect, however, could be more widespread. For example, it has been reported that ALDH1L1 could alter the overall flux of folate-bound one-carbon groups and thus cellular methylation [30]. In addition, *Aldh1l1* KO mice demonstrated that the loss of the enzyme induces a functional folate deficiency because the THF pool cannot be replenished from the 10-formyl-THF conversion [36]. Of note, dietary folate deficiency is a risk factor for carcinogenesis (reviewed in [80,81]). In this regard, we have previously reported that dietary folate restriction activates Rac1 [82], a Rho GTPase which has been shown to promote DEN-induced liver tumors [83]. Another mediator that might link ALDH1L1 to DEN-induced carcinogenesis is Bid, a proapoptotic member of the Bcl-2 protein family [84]. It has been reported that Bid may promote hepatic carcinogenesis via the control of growth and inflammatory responses [85], but at the same time, Bid itself is regulated by ALDH1L1 through the JNK-dependent mechanism, controlling its stability [47].

ALDH1L1 may contribute significantly to NADPH production. Though such contribution was not evaluated in our study, it has been reported that a related mitochondrial folate enzyme, ALDH1L2, is responsible for the production of a significant portion of mitochondrial NADPH [17]. Through NADPH production, ALDH1L1 can affect an even larger network of metabolic pathways. Of note, NADPH is required to maintain cellular antioxidants, most importantly GSH, in a reduced state. This also would explain alterations in GSH levels associated with the ALDH1L1 loss. To this end, it has been reported that ALDH1L1 is involved in balancing oxidative stress [86]. DEN induces carcinogenesis by the formation of DNA adducts [34], a mechanism seemingly not linked to ALDH1L1-related pathways. These findings are consistent with our observation that ALDH1L1 does not appear to influence early carcinogenesis and tumor initiation. Interestingly, however, a dramatic elevation of ADP-ribose was observed in our study in WT mice between 10 and 20 weeks after DEN treatment, which we interpret as the attempt of liver cells to repair DNA damage. Indeed, PARP activation is an immediate cellular response to metabolic, chemical, or radiation-induced DNA single-strand breaks [87]. Such ADP-ribose elevation was weakened by the lack of ALDH1L1, suggesting some effect of the enzyme on DNA repair. More relevant to the ALDH1L1 function and the ALDH1L1-associated cellular GSH status, however, it has been shown that DEN-induced carcinogenesis is associated with increased oxidative stress and inflammation [57,88,89]. In further support of the ALDH1L1 link to this mechanism, our previous studies of *Aldh1l1* KO mice showed that the enzyme influences the expression of inflammation-related genes [36].

## 5. Conclusions

Overall, the role of ALDH1L1 in carcinogenesis is likely complex and not necessarily unidirectional. While the ALDH1L1 protein is commonly lost in malignant human tumors, it is not clear whether the lack of corresponding enzymatic activity can promote tumorigenesis. Our study suggests that ALDH1L1 participates in the regulation of the proliferation of HCC but not in the initiation of DEN-induced liver carcinogenesis. Although the effect of ALDH1L1 on tumorigenesis could be cancer type-specific, and the possibility that the enzyme interferes with malignant transformation still cannot be excluded, the present state of knowledge supports the role of the enzyme as a proliferation suppressor in formed malignant lesions. Reports that ALDH1L1 protein is more strongly downregulated in advanced cancers [8,9,26] are in agreement with the effect of the enzyme on proliferation rather than initiation. Importantly, we showed that the downregulation of ALDH1L1 expression in advanced DEN tumors developed in wild-type mice, which experimentally confirmed the selective proliferative advantage of the loss of this enzyme. Our study also highlighted metabolic pathways that are likely to differentially mediate the response to DEN depending on the presence of the ALDH1L1. Although the specific contribution of each of these pathways in DEN-induced carcinogenesis and their regulation by ALDH1L1 requires further investigation, the enzyme most directly regulated THF biosynthesis (Figure 5). Mechanistically, this pathway is linked to glycine generation from serine, and our metabolomic studies provide evidence that the effect on glycine levels drives key downstream metabolic pathways involved in tumor progression, including the glutathione cycle (Figure 9). Indirectly, ALDH1L1 status also affected glucose metabolism (Figure 8). We link this phenomenon to a more general effect of ALDH1L1 on folate metabolism, in which deregulation was shown to affect levels of glucose and glucose-related pathways [90,91]. It should be noted that, based on reported genomic and transcriptomic profiles, the DEN model of hepatocellular carcinogenesis might not be the model that most accurately reflects HCC-related processes in humans [92]. However, despite this limitation, in our study, this model provides further insight into the role of the ALDH1L1 protein as a regulator of cellular proliferation and a putative tumor suppressor.

## Data Availability

The data presented in this study are available in this article and Supplementary Materials.

## Supplementary Materials

The following are available online at https://www.mdpi.com/article/10.3390/cancers13133219/s1, Figure S1: Liver weight normalized to body weight for Aldh1l1^+/+^ (WT) and Aldh1l1^−/−^ (KO) male mice injected with DEN, Figure S2: H&E (upper panels) and reticulin staining (lower panels) of Aldh1l1^+/+^ (WT) and Aldh1l1^−/−^ (KO) mouse liver after DEN injection (weeks), Figure S3: Whole WT and KO mouse livers imaged at indicated time points post-DEN injection, Figure S4: Vascular density was evaluated using immunohistochemistry for CD34, Figure S5: Acylglycine conjugates identified by metabolomic approach, Figure S6: Fatty acids identified by metabolomic approach, Figure S7: Images of full blots for Figure 1G, Table S1: Primers used for genotyping, Table S2: Primers used for real-time PCR, Table S3: Metabolomics data, Table S4: VIP values. The uncropped blots are shown in Supplementary Materials.
