# Supplementary material for: Knockout of Putative Tumor Suppressor Aldh1l1 in Mice Reprograms Metabolism to Accelerate Growth of Tumors in a Diethylnitrosamine (DEN) Model of Liver Carcinogenesis
Source: Cancers (Basel). 2021 Jun 28;13(13):3219. doi: 10.3390/cancers13133219 (PMC8268287; doi:10.3390/cancers13133219)
Supplement: Supplementary file 1 [file cancers-13-03219-s001.zip › Supplementary/cancers-1257624 Figure S7 uncropped blot (R1 Full Blot) .pdf]

Krupenko et al.
Manuscript ID:
Cancers-1060009

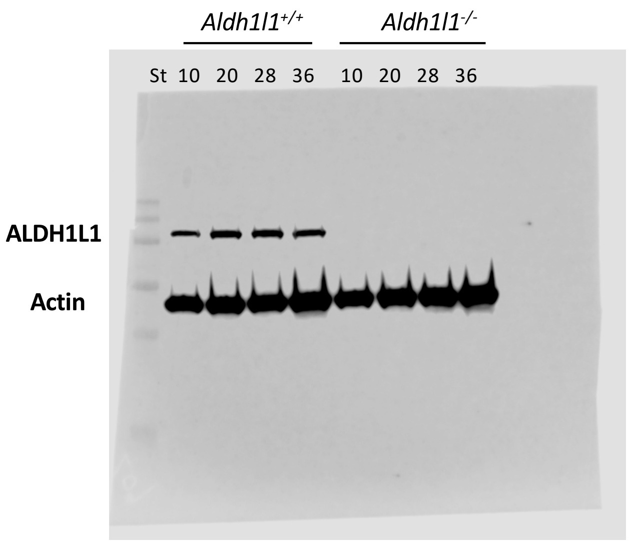

2 min exposure

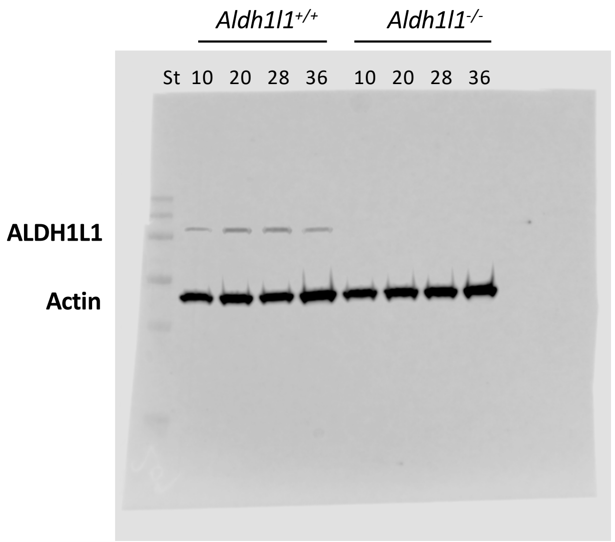

30 s exposure

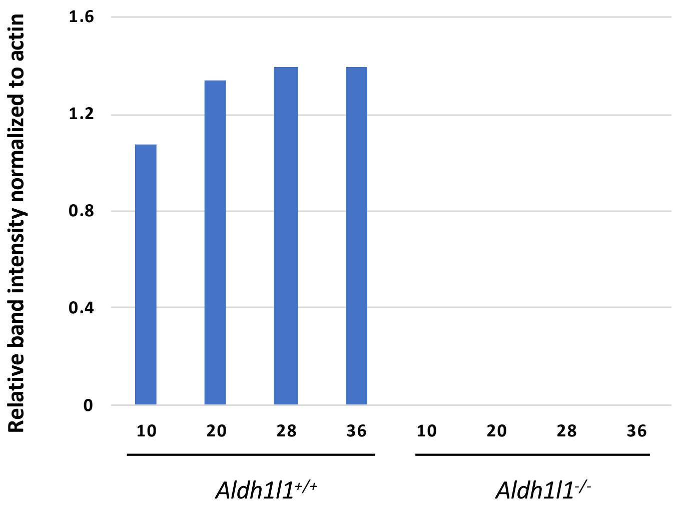

Images of full blots for Fig. 1F of the main manuscript are shown (two exposure times were used, 2 min and 30 s). *Bottom panel* shows intensity of bands assessed using Image J. Numbers on blots and graph show weeks post-DEN injection. *St*, molecular mass standards.
