# Supplementary material for: Knockout of Putative Tumor Suppressor Aldh1l1 in Mice Reprograms Metabolism to Accelerate Growth of Tumors in a Diethylnitrosamine (DEN) Model of Liver Carcinogenesis
Source: Cancers (Basel). 2021 Jun 28;13(13):3219. doi: 10.3390/cancers13133219 (PMC8268287; doi:10.3390/cancers13133219)
Supplement: Supplementary file 1 [file cancers-13-03219-s001.zip › Supplementary/Supplementary Tables and Figures of Cancers-1257624.pdf]

Supplementary Materials (Krupenko et al)

1. Tables S1 and S2

2. Figures S1–S6

3. Table S3 and S4 (separate Excel files)

Table S1. Primers used for genotyping.

Primers	Sequence	PCR fragment (bp)
Forward for WT allele	5′- CCGAAGGACCAAACTCTTTCC-3′	199 bp
Reverse for WT allele	5′- TCCTTACAGCCCTTTCCAGAG-3′	
Forward for KO allele	5′- CACACCTCCCCCTGAACCTGAAA -3′	685 bp
Reverse for KO allele	5′- AAGCTTCCTCTCTGTTCTACCCACAC-3′	

Table S2. Primers used for real-time PCR.

Gene	Forward primer	Reverse primer
Bax	CGG CGA ATT GGA GAT GAA CTG	GCA AAG TAG AAG AGG GCA ACC
Bclxl	AGG TTC CTA AGC TTC GCA ATT C	TGT TTA GCG ATT CTC TTC CAG G
CyclinD1	CAG AGG CGG ATG AGA ACA AG	GTT GTG CGG TAG CAG GAG AG
CyclinE1	TCC GAC CTT TCA GTC CGC TC	GGG GAT GAA AGA GCA GGG GT
Gdf11	GAG TAC CAC GCT ACC ACC GA	CAC CCA CAG TTG GGC CTT CA
H_2ax	CCG GCG GTC GGC AAG	AAG TGG CTC AGC TCT TTC TGT GAG
IL-1	AGT TGC CTT CTT GGG ACT GA	TCC ACG ATT TCC CAG AGA AC
IL-10	TCG GCC AGA GCC ACA TG	TTA AGG AGT CGG TTA GCA AGT ATG TTG
IL-6	CTG CAA GAG ACT TCC ATC CAG	AGT GGT ATA GAC AGG TCT GTT GG
Ki67	GTC CTC GGC TCA CCT GGT C	TGA CAC TAC AGG CAG CTG GA
NFKb	TGA GAA GAA CAA GAA ATC CTA CCC A	GTG CAT ACC CCG TCC TCA CA
p21	GTC AGG CGC AGA TCC ACA G	AAG TTC CAC CGT TCT CGG GC
p53	CCG AAG ACT GGA TGA CTG CCA	ACT CGG AGG GCT TCA CTT GG
PCNA	TAG CCA TGG GCG TGA ACC TC	TCT GGG ATT CCA AGT TGC TCC A
TGFB	CTG ACC CCC ACT GAT ACG CC	GCG CTG AAT CGA AAG CCC TG
TGFBR	CTG ACC CCC ACT GAT ACG CC	GCG CTG AAT CGA AAG CCC TG
TNF-α	CAG GCG GTG CCT ATG TCT C	CGA TCA CCC CGA AGT TCA GTA G
VEGF	TGC ACC CAC GAC AGA AGG AG	TCG TCG GGG TAC TCC TGG AA
GAPDH	TGC CCC CAT GTT TGT GAT G	TGT GGT CAT GAG CCC TTC C

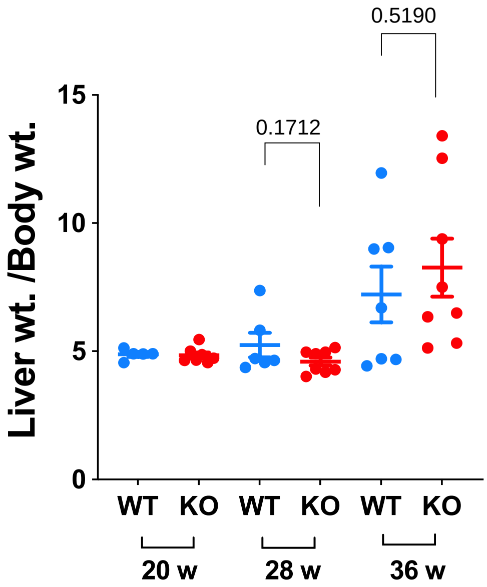

Figure S1. Liver weight normalized to body weight for Aldh1l1$^{+/+}$ (WT) and Aldh1l1$^{-/-}$ (KO) male mice injected with DEN. Time post-DEN injection (weeks) and p value are shown.

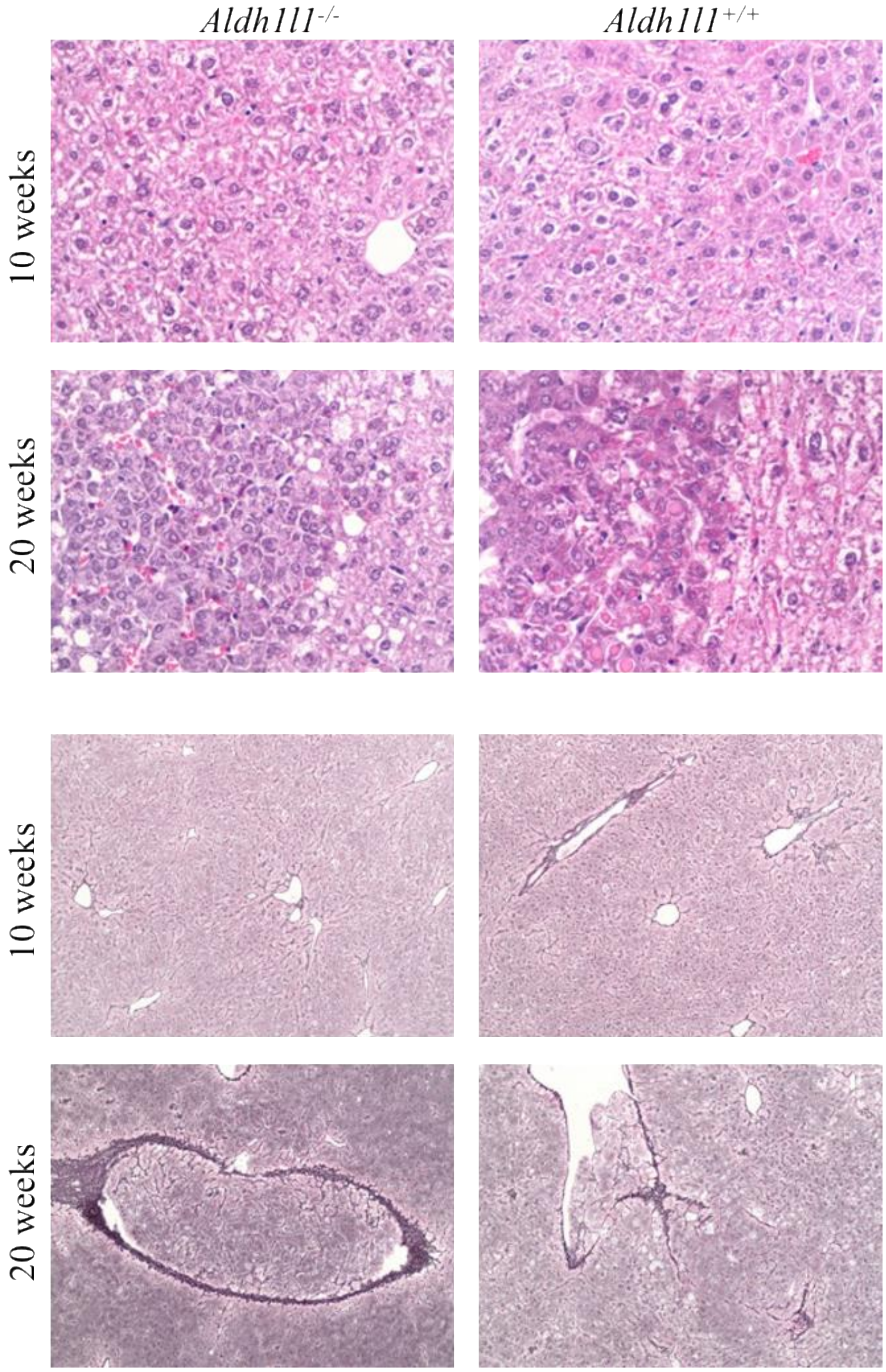

Figure S2. H&E (*upper panels*) and reticulin staining (*lower panels*) of *Aldh1l1*$^{+/+}$ (WT) and *Aldh1l1*$^{-/-}$ (KO) mouse liver after DEN injection (weeks).

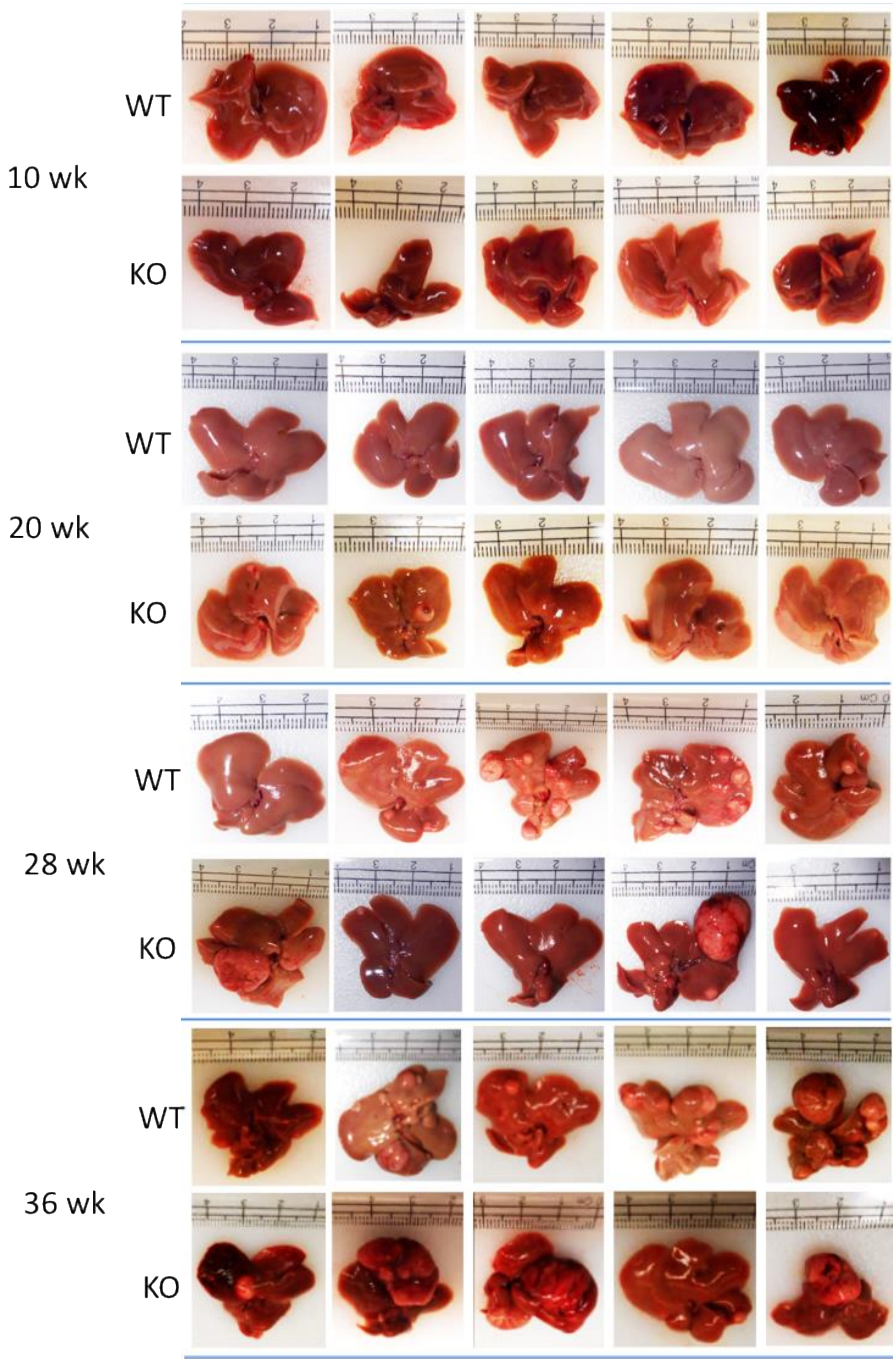

Figure S3. Whole WT and KO mouse livers imaged at indicated time points post-DEN injection. Macroscopic tumors are visible starting 20-week time point in KO. (*Aldh1l1*$^{-/-}$) and 28-week time point in WT (*Aldh1l1*$^{+/+}$) mice. Livers from five mice in each group are shown.

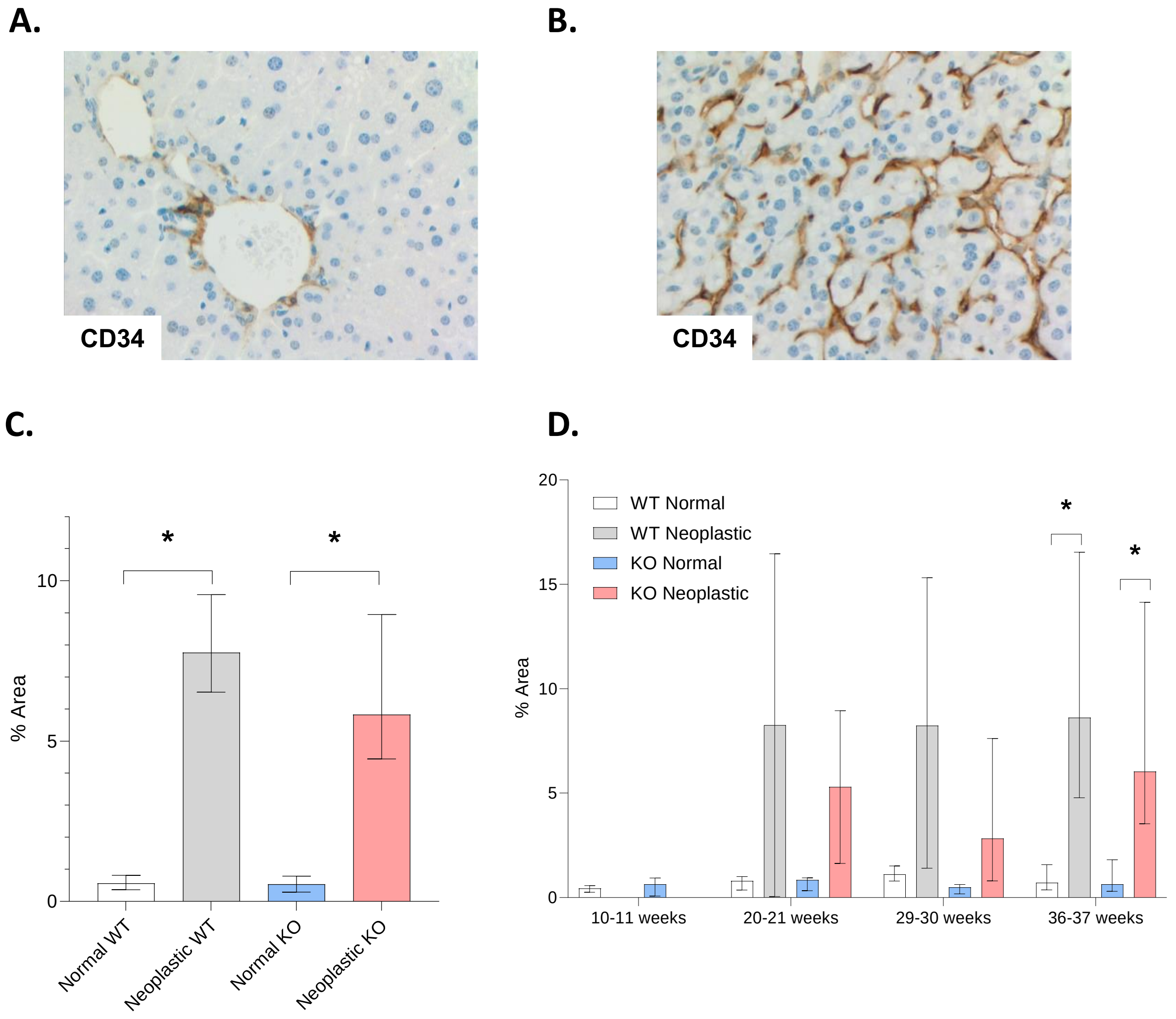

Figure S4. Vascular density was evaluated using immunohistochemistry for CD34. (A) Normal liver demonstrated CD34 staining surrounding central and portal vessels. (B) Neoplastic liver nodules demonstrated CD34-staining in a sinusoidal pattern, illustrating increased vascular density. (C) Vascular density was evaluated by examining photomicrographs taken at 40x magnification. ImageJ software was used to count the number of stained pixels in each Image and determine % area stained. At 36-37 weeks, neoplastic nodules demonstrated significantly increased capillary density compared to normal liver. We observed no difference in vascular density between KO and WT neoplastic nodules.

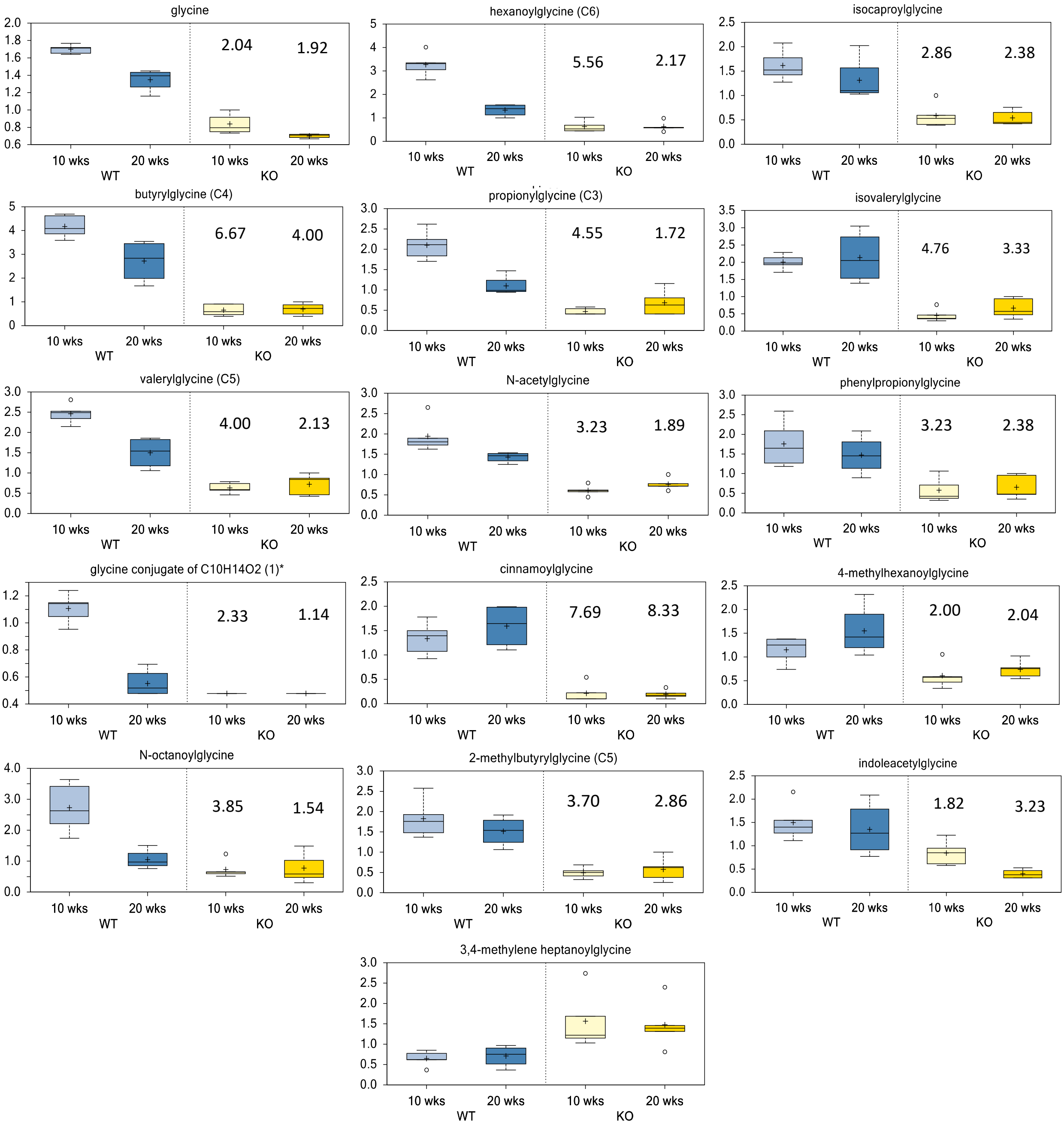

Figure S5. Acylglycine conjugates identified by metabolomic approach.

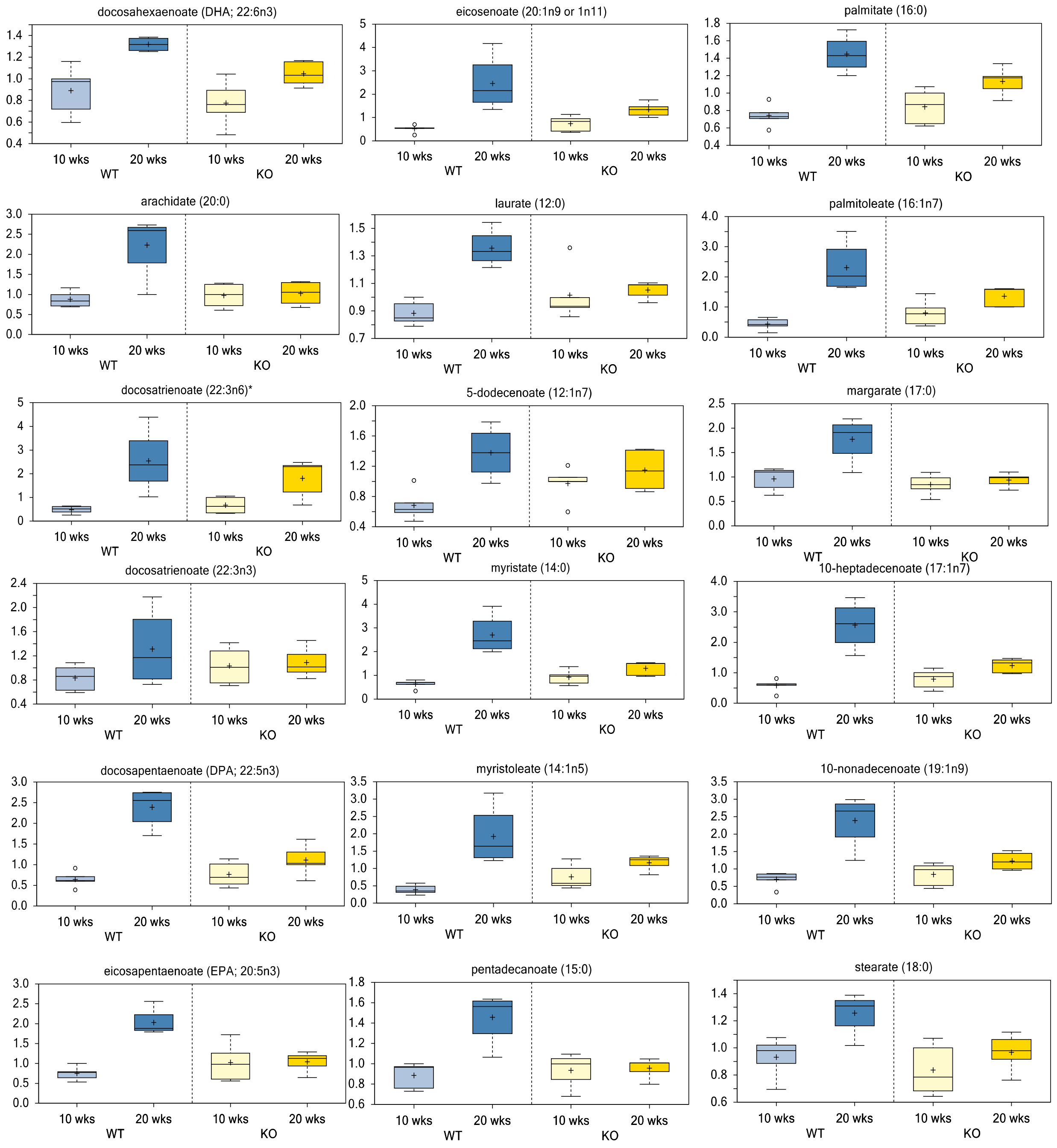
docosahexaenoate (DHA; 22:6n3)
eicosenoate (20:1n9 or 1n11)
palmitate (16:0)
arachidate (20:0)
laurate (12:0)
palmitoleate (16:1n7)
docosatrienoate (22:3n6)*
5-dodecenoate (12:1n7)
margarate (17:0)
docosatrienoate (22:3n3)
myristate (14:0)
10-heptadecenoate (17:1n7)
docosapentaenoate (DPA; 22:5n3)
myristoleate (14:1n5)
10-nonadecenoate (19:1n9)
eicosapentaenoate (EPA; 20:5n3)
pentadecanoate (15:0)
stearate (18:0)
10 wks
20 wks
WT
KO

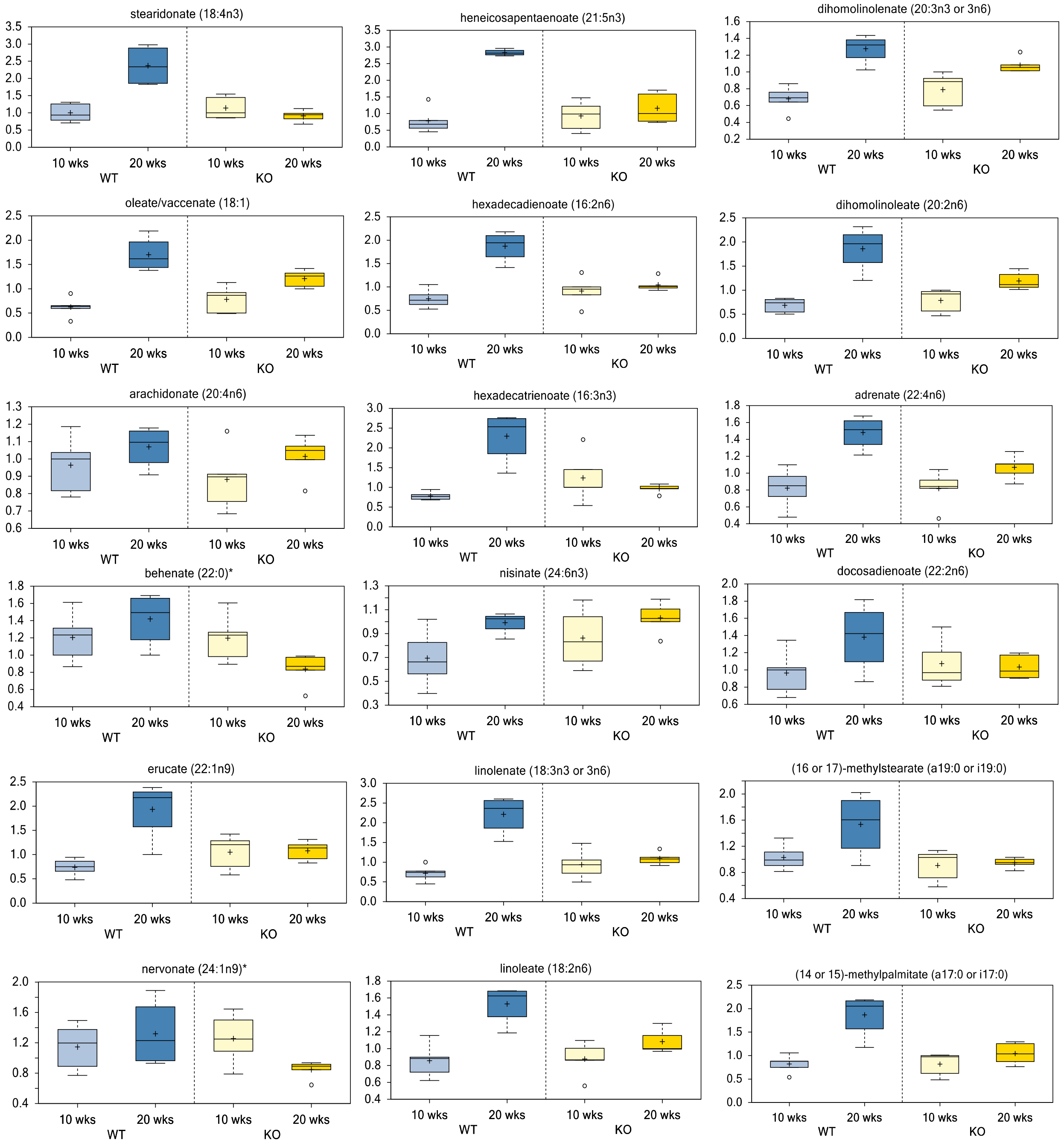

Figure S6. Fatty acids identified by metabolomic approach.
